# Beyond Reading Modulation: Temporo-Parietal tDCS Alters Visuo-Spatial Attention and Motion Perception in Dyslexia

**DOI:** 10.3390/brainsci11020263

**Published:** 2021-02-19

**Authors:** Giulia Lazzaro, Sara Bertoni, Deny Menghini, Floriana Costanzo, Sandro Franceschini, Cristiana Varuzza, Luca Ronconi, Andrea Battisti, Simone Gori, Andrea Facoetti, Stefano Vicari

**Affiliations:** 1 Child and Adolescent Psychiatry Unit, Department of Neuroscience, Bambino Gesù Children’s Hospital, IRCCS, 00146 Rome, Italy; giulia.lazzaro@opbg.net (G.L.); deny.menghini@opbg.net (D.M.); floriana.costanzo@opbg.net (F.C.); cristiana.varuzza@opbg.net (C.V.); andrea.battistidys@gmail.com (A.B.); 2 Department of Human Science, LUMSA University of Rome, 00193 Rome, Italy; 3 Department of Human and Social Science, University of Bergamo, 24129 Bergamo, Italy; sara.bertoni@unibg.it (S.B.); simone.gori@unibg.it (S.G.); 4 Department of General Psychology, University of Padova, 35131 Padova, Italy; sandro.franceschini@unipd.it (S.F.); andreafacoetti@unipd.it (A.F.); 5 School of Psychology, University “Vita-Salute San Raffaele”, 20132 Milano, Italy; ronconi.luca@unisr.it; 6 Department of Life Sciences and Public Health, Catholic University of the Sacred Heart, 00168 Rome, Italy

**Keywords:** neuromodulation, brain reading networks, magnocellular-dorsal pathway, attention, neural noise, cortical excitability, children and adolescents

## Abstract

Dyslexia is a neurodevelopmental disorder with an atypical activation of posterior left-hemisphere brain reading networks (i.e., temporo-occipital and temporo-parietal regions) and multiple neuropsychological deficits. Transcranial direct current stimulation (tDCS) is a tool for manipulating neural activity and, in turn, neurocognitive processes. While studies have demonstrated the significant effects of tDCS on reading, neurocognitive changes beyond reading modulation have been poorly investigated. The present study aimed at examining whether tDCS on temporo-parietal regions affected not only reading, but also phonological skills, visuo-spatial working memory, visuo-spatial attention, and motion perception in a polarity-dependent way. In a within-subjects design, ten children and adolescents with dyslexia performed reading and neuropsychological tasks after 20 min of exposure to Left Anodal/Right Cathodal (LA/RC) and Right Anodal/Left Cathodal (RA/LC) tDCS. LA/RC tDCS compared to RA/LC tDCS improved text accuracy, word recognition speed, motion perception, and modified attentional focusing in our group of children and adolescents with dyslexia. Changes in text reading accuracy and word recognition speed—after LA/RC tDCS compared to RA/LC—were related to changes in motion perception and in visuo-spatial working memory, respectively. Our findings demonstrated that reading and domain-general neurocognitive functions in a group of children and adolescents with dyslexia change following tDCS and that they are polarity-dependent.

## 1. Introduction

Dyslexia is a heritable neurodevelopmental disorder characterized by persistent difficulties with accurate or fluent word recognition [1] that relies on multi-faceted brain dysfunction [2,3].

Replicated structural/functional neuroimaging studies have demonstrated specific neural signatures for dyslexia. Neuroimaging findings have converged on atypical activation in a distributed left-hemisphere network in children and adults with dyslexia [4]. As identified by meta-analyses, the most consistent finding is dyslexic hypoactivation relative to typical readers in the left temporo-occipital regions—critical for the automatic visual processing of word strings or print [5]—and in the left temporo-parietal regions—important for grapheme-to-phoneme mapping [6]. Evidence showed that children with dyslexia exhibit hypoactivation in the bilateral temporo-parietal regions, not only compared to chronological age-matched groups, but also to reading-matched children [7], excluding, therefore, the contribution of reading experience to these hypoactivations. The reduced activation of the bilateral temporo-parietal regions—including the left inferior parietal cortex close to the intraparietal sulcus [5]—could indicate a possible role of the dorsal attentional network dysfunction [8] in dyslexia and highlights the contribution of domain-general neurocognitive functions in reading acquisition [9].

At the same time, clinical [10], longitudinal [11,12,13,14,15,16], intervention [17,18], and illiterate vs. literate populations [19] studies suggest that the typical development of the temporo-occipital and the temporo-parietal reading networks require the efficiency of multiple domain-general neurocognitive functions, confirming the interactive specialization of reading circuits during the development [20].

Various neuropsychological theories have tried to explain deficits related to dyslexia. Among these, the most influential is the phonological core deficit theory [16,17,21,22,23], which argues that dyslexia stems from deficits in the ability to identify and explicitly act upon sounds of spoken words, leading to difficulties in learning appropriate grapheme-to-phoneme mapping. Longitudinal studies had shown that phonological awareness and phonological working memory, as well as rapid automatized naming (RAN), appear good predictors of future reading development [11,15,16,17,23]. Intriguingly, comparisons between illiterate and literate individuals on phoneme awareness, verbal working memory, and RAN ability suggest that these phonological skills might be influenced by emergent literacy [24,25,26,27]. Moreover, a recent study confirms that both short-term and long-term verbal memory deficits in children with dyslexia could be explained by reduced reading experience [28].

Multiple domain-general neurocognitive functions, such as rapid auditory sensory processing [29,30,31] and auditory and visual selective attention [11,18,32,33,34,35,36,37], as well as motion perception—indexed by performance on coherent dot motion (CDM) task, and specifically processed by the magnocellular–dorsal (MD) stream [10,12,38,39,40,41,42,43], have been widely recognized as correlates of dyslexia [44,45,46,47,48,49]. A multisensory sluggish attentional shifting—induced by an MD stream dysfunction [32]—might be linked to the impairments of verbal and visual-spatial working memory typically shown in children with dyslexia [50].

Transcranial direct current stimulation (tDCS) is showing great promise as a tool for manipulating neural activity and, in turn, multiple neurocognitive processes. The basic idea of tDCS is that the application of a weak current can interact with ongoing brain functioning and modify plasticity, and that this, in turn, can modulate the behavior [51]. tDCS is based on the application of a continuous, low-intensity electrical current, where one typically observes patterns of excitatory/inhibitory modulation depending on the polarity used [52] in the target area under the electrodes as well as in distant brain regions [53]. The direction of polarization depends strictly on the orientation of axons and dendrites of the brain region targeted by the induced electrical field, but anodal stimulation generally increases excitability on the target area while cathodal stimulation reduces excitability [52].

A number of studies have demonstrated the positive effect of tDCS on reading [54,55,56] and, particularly, in dyslexia [57,58,59,60,61,62]. In typically reading adults, an overall improvement in word reading efficiency has been observed after the application of left anodal/right cathodal tDCS over the temporo-parietal areas [54] as well as following left anodal stimulation over the inferior parietal lobule [55,56]. In adults with dyslexia, an enhancement in text reading fluency was demonstrated after left anodal stimulation over the visual extrastriate area—a region commonly implicated in the magnocellular system [57]. In children with dyslexia, a substantial effect on word reading fluency [62], low-frequency word reading accuracy, and nonword reading fluency [59,60] was found after six weeks of left anodal/right cathodal tDCS over the temporo-parietal regions. Similarly, a beneficial effect on text and nonword reading accuracy was shown following five sessions of left anodal tDCS over the mid-posterior temporal area in children and adolescents with dyslexia [61].

However, the multiple domain-general neurocognitive changes beyond reading modulation have been poorly investigated across studies.

To the best of our knowledge, only one study in children and adolescents with dyslexia [58] examined changes in reading-related functions. The study [58] aimed at investigating whether tDCS over temporo-parietal regions could affect reading but also phonological processing (phonemic blending), verbal working memory (verbal n-back), and RAN in a polarity-dependent way. In this within-subjects design, nineteen children and adolescents with dyslexia were evaluated before tDCS and immediately after 20 min of exposure to three different tDCS conditions: (i) left anodal/right cathodal (LA/RC) tDCS, (ii) right anodal/left cathodal (RA/LC) tDCS, and (iii) sham tDCS. A significant reduction in text reading errors after LA/RC tDCS and an increase in errors after RA/LC tDCS were found, whereas changes did not emerge in phonological skills.

tDCS studies that examine not only reading and phonological processing, but also MD stream-related functions and visual-spatial attention are needed to better understand how and to what extent temporo-parietal brain modulation alters the multiple general neurocognitive domains that support reading development [11,14,33,35,44,48].

The aim of the present study was to examine whether, in addition to reading, can MD stream functioning and visuo-spatial attention also change after temporo-parietal tDCS in children and adolescents with dyslexia, and whether these changes are polarity-dependent.

For this purpose, ten participants with dyslexia performed reading and neuropsychological tasks (i.e., phonemic blending, verbal and visuo-spatial n-back, letters and colors RAN, attentional zooming and CDM task) after 20 min of exposure to LA/RC tDCS and to RA/LC tDCS over temporo-parietal regions.

## 2. Materials and Methods

### 2.1. Participants

The study included ten children and adolescents with dyslexia (8 females; age range: 10.08–16.67 years, age mean ± standard deviation (SD): 13.89 ± 2.4 years). Participants had a nonverbal intelligence quotient [63,64] in the normal range (Intelligence quotient mean ± SD: 103.70 ± 12.26). All children and adolescents were native Italian speakers with normal or corrected-to-normal vision and were right-handed according to the Edinburgh Handedness Inventory [65]. Participants were a subgroup of children tested by Costanzo et al. [58].

The diagnosis of dyslexia was based on the DSM-5 criteria [1] and was made with a comprehensive diagnostic battery, including word and pseudoword lists [66,67] and text reading tasks [68,69]. Each participant was included in the study when the speed or accuracy in text and/or word and/or nonword reading tasks was at least 1.5 SDs below the population mean for school-age. Participants with a personal history of neurological disease or a family history of epilepsy and a comorbidity with attention deficit or hyperactivity disorder as assessed by clinical examination and by the Conners’ Rating Scales—Revised [70] were excluded.

Each participant was evaluated at the Child and Adolescent Psychiatry Unit of the Bambino Gesù Children’s Hospital by a team of expert clinicians, including a Psychologist, a Neurologist, and a Speech Therapist.

Written informed consent was obtained from all participants and their parents after the procedures had been fully explained. This study was carried out in accordance with the World Medical Association’s Declaration of Helsinki and was approved by the Research Ethical Committee of the Bambino Gesù Children’s Hospital (process number 201201X002931).

### 2.2. Experimental Design

All participants were exposed to two tDCS conditions with a minimum intersession interval of 24 h: (i) LA/RC tDCS and (ii) RA/LC tDCS to investigate the effect of left or right lateralization of temporo-parietal regions on reading, phoneme blending, verbal and visuo-spatial working memory, MD stream, and visuo-spatial attention functioning. The order of conditions was counterbalanced between participants. An examiner, who was blinded to the identity of the conditions, assessed reading, phonological, and multiple neurocognitive measures after each tDCS session (when electrodes were removed). For each session (the two stimulation conditions), motion perception and visuo-spatial attention tasks, as well as two different sets of reading and neuropsychological tasks were presented immediately after 20 min of tDCS.

### 2.3. Stimulation Conditions

A direct current was generated by a BrainStim Stimulator AQ5 (E.M.S. s.r.l.; Bologna, Italy) and delivered through a pair of identical, square, scalp electrodes (5 × 5 cm) of conductive rubber and covered with saline-soaked synthetic sponges. Electrodes were positioned over the left and right temporo-parietal regions according to the 10–20 EEG on the sites corresponding midway between P7 and TP7 and midway between P8 and TP8, respectively. In the LA/RC condition, the anode was placed over the left temporo-parietal regions, whereas the cathode was placed over the contralateral regions; conversely, in the RA/LC condition, the anode was placed over the right temporo-parietal regions and the cathode was placed over the contralateral regions. As a reference electrode site, we chose to exclude other brain regions, such as the prefrontal and the occipital cortices, to not affect mechanisms that might relate to reading processes.

At the beginning of both LA/RC and RA/LC conditions, the current was increased slowly during the first 30 s (ramp-up) to 1 mA and the current was decreased slowly to 0 mA during the last 30 s (ramp-down). Between the ramp-up and the ramp-down, a constant direct current (1 mA) was delivered for 20 min.

### 2.4. Reading and Neuropsychological Tasks

#### 2.4.1. Words, Pseudowords, and Text Reading

Every session included a set of words or pseudowords to read aloud. Specifically a set of 20 high frequency words (HF; 10 trisyllabic such as divano = sofa, famiglia = family; 10 disyllabic such as: treno = train, anno = year); 20 low frequency words (LF; 10 trisyllabic such as: calcagno = heel, bussola = compass; 10 disyllabic such as: bava = burr, urna = urn); 20 pseudowords (PW; 10 trisyllabic such as: badoma; 10 disyllabic such as: espa), created by rearranging the character-string of real word items; and a text 215 words long (about 400 syllables long). Stimuli were taken from a modified version of those adopted in Costanzo and colleagues’ studies [71,72].

For scoring of reading errors, one point was assigned for each letter substitution (i.e., errors that involved consonant or vowel sound changes, omissions, position changes, or additions) and half point for every self-correction and hesitation. No more than one error point was assigned for the same item.

TEXT reading errors were calculated as a percentage of the total errors on the total number of words in the text (% of errors), while TEXT reading times were calculated by dividing the total time in seconds for text reading by the total number of word syllables and multiplying by 100. In HF, LF, and PW tasks, reading errors were calculated as the percentage of total errors on the total number of words of each list (% of errors), while reading times were the total time in seconds to read the list.

#### 2.4.2. Lexical Decision

The task required participants to classify 20 strings of letters visually presented on the computer monitor as words or pseudowords. The median reaction times (RTs) in milliseconds (ms) of the correct responses were computed and considered for the statistical analyses. RTs beyond 2SDs of the individual mean were omitted, based on the assumption that these responses could have involved attentional lapses or blinks.

#### 2.4.3. Phoneme Blending

Participants had to hear all the phoneme sounds and put the sounds together to make a pseudoword. Each session included a set of 10 pseudowords. The number of phonemes correctly blended (Number of Phonemes) and the total time in seconds for each phoneme (Phonemes Times) were calculated and considered.

#### 2.4.4. Working Memory

The N-back working memory tasks were composed of verbal and the visuo-spatial conditions.

The verbal condition (Verbal N-back) consists of listening to a continuous stream of letters. After a training phase, participants are required to decide whether each letter matches to the letter heard immediately before (level: 1-back). The visuo-spatial condition (Visuo-Spatial N-back) consists of presenting a series of visual stimuli (black boxes) in a certain location on the screen. After a training phase, participants are required to indicate whether the location of each box presented matches to the location of the box presented immediately before (level: 1-back).

In each condition, a total of 35 trials with an interval stimulus of 3.20 s was presented. The number of correct answers and errors was considered and was used to calculate the percentage of accuracy of each level of n-back. For both conditions, when the accuracy was equal to or more than 80%, the evaluator increased the difficulty of the n-back (for example, passing from 1-back to 2-back). For both conditions, an index of efficiency was calculated as the number of the last n-back achieved (i.e., percentage accuracy value ≥ 80%) followed by the percentage of the accuracy of the unachieved n-back (i.e., percentage accuracy value < 80%). For example, if a child achieves the 2-back level but fails at the 3-back level with a percentage of accuracy of 60%, the index of efficiency is 2.60.

#### 2.4.5. Rapid Automatized Naming

RAN was measured by two tasks: the letter-naming and the color tasks [73]. Eight letters and eight colored circles were printed in two lines on a white sheet of A4 paper. Participants had to name aloud the stimuli on the sheet as soon as possible. In each task, the total time in seconds was considered for the analysis.

#### 2.4.6. Coherent Dot Motion

**Figure 1:**
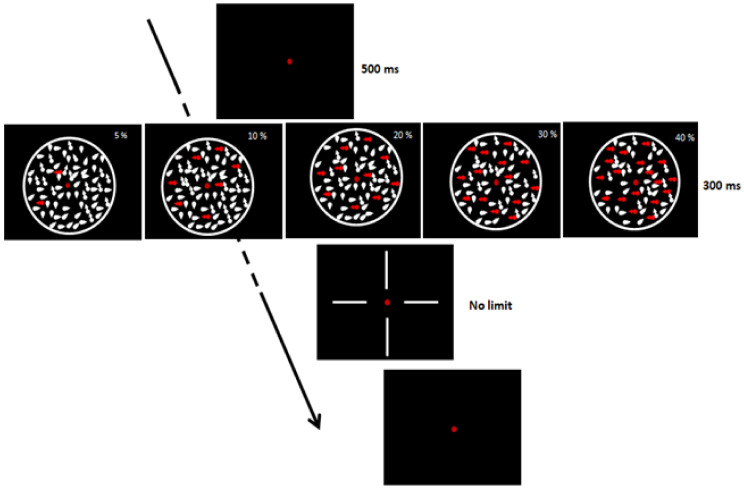
Coherent dot motion task.

Participants had to discriminate the direction of dot movement (upward, downward, left, or right; chance level = 0.25), and only response accuracy was collected. There were 5 levels of coherence, randomly intermixed (5, 10, 20, 30, and 40%). The experimental session consisted of 100 trials (20 trials for each coherence level). The CDM display duration was 300 ms (Figure 1). Participants were seated in a dimly lit room in front of a 15-in CRT monitor placed at a viewing distance of 57 cm (screen resolution 1024 × 768/60 Hz, with 0.3 mm pixel size). After the fixation point (a red dot in the center of the screen (500 ms)), white dots, subtending a visual angle of 0.08°, appeared on a black background. Dots were contained in a circle of 13° in diameter and their number was approximately 10 deg−2 at each frame (duration = 16.7 ms). The dots’ density remained constant throughout the trial using the Shadlen–Movshon algorithm with a limited lifetime of 3 frames [74,75]. The dots’ speed was 7°/s. The procedure was similar to the one adopted by Ronconi et al. [76].

#### 2.4.7. Attentional Zooming

**Figure 2:**
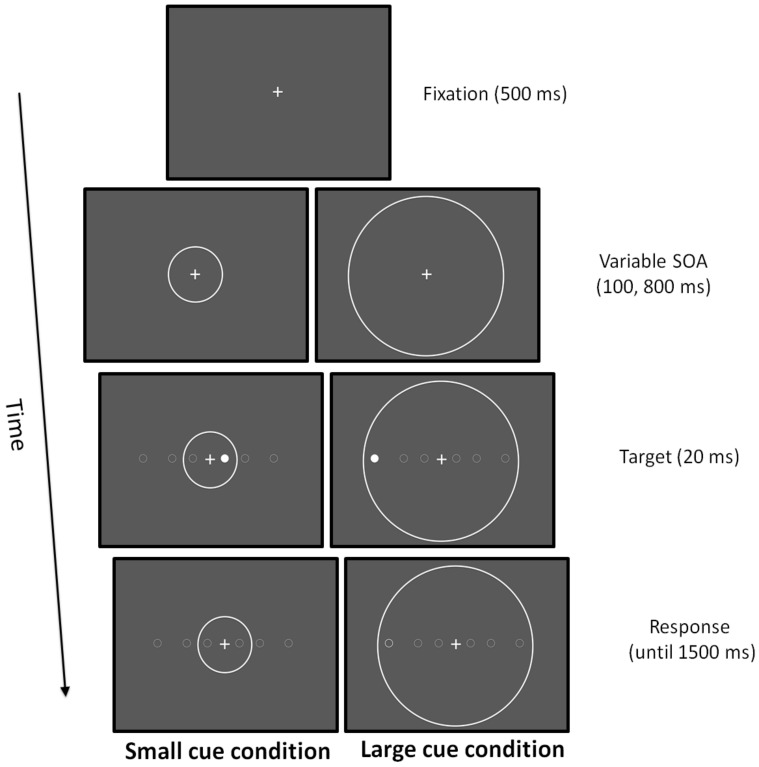
Attentional zooming task.

Participants were instructed to keep their eyes on the fixation point throughout the duration of the trial and had to press a button on the keyboard as fast as possible to detect a simple target that was randomly presented in the left and in the right visual hemi-field. The target stimulus was a dot of 0.5 deg that could appear at three possible horizontal eccentricities (2, 6, and 12 deg). Before the target, two cue conditions could be presented: in the small cue condition, a circle with a ray of 4 deg was presented concentrically to the fixation point; in the large cue condition, a circle with a ray of 12.5 deg was presented concentrically to the fixation point. In the small cue condition, the target was displayed inside the focusing cue at 2 deg, whereas at 6 and 12 deg, it appeared outside. In the large cue condition, the target was always displayed inside the focusing cue [77,78]. Stimuli were light grey displayed on a black background. Participants were seated in a dimly lit room in front of a 15-in CRT monitor placed at a viewing distance of 50 cm. The fixation point, a cross of 0.5 deg, was presented for 500 ms. Subsequently, the cue was presented and after a stimulus onset asynchrony (SOA) of 100 or 800 ms, the target was displayed for 20 ms. If any response was given after 1500 ms, a new trial was presented. The cue was displayed until the response of the participant (Figure 2). Experimental trials were, in total, 132:120 real trials and 12 catch trials where no target was presented to prevent anticipatory responses. RTs were collected.

### 2.5. Statistical Analyses

The Shapiro–Wilk test was used to test the normality of the data and Levene’s test for the homogeneity of variances. When data were normally distributed and the assumption of homogeneity was not violated, parametric analyses were computed. When one assumption was not met, non-parametric tests were conducted.

Wilcoxon signed-rank tests were used to analyze reading changes after LA/RC vs. RA/LC stimulations, and also to verify changes after LA/RC vs. RA/LC stimulations in the following scores: Number of Phonemes, Phonemes Times, Verbal N-back, Visuo-Spatial N-back, Letters RAN, and Colors RAN scores.

To evaluate motion perception differences after stimulation conditions in the CDM task, a repeated measures ANOVA was conducted with Response Accuracy as the dependent variable and Condition (LA/RC vs. RA/LC) and Levels of Coherence (5, 10, 20, 30, and 40%) as within-subject factors.

To evaluate attentional zooming differences after stimulations in the Attentional Zooming Task, a repeated measures ANOVA was computed with RTs as the dependent variable and Condition (LA/RC vs. RA/LC), Cue Size (Small vs. Large), Target Eccentricity (2, 6, and 12 deg), Hemi-field of target presentation (Left vs. Right), and SOA (100 vs. 800 ms) as within-subject factors.

Post hoc analyses were performed by using a *t*-test. Partial eta squared (ηp^2^) and Cohens’ d were used as measures of effect sizes.

To verify the relation between reading and neuropsychological changes, Spearman’s correlations were computed.

A *p* value ≤ 0.05 was considered statistically significant.

## 3. Results

Table 1 depicts the mean (SD) of each reading measure and lexical decision task for LA/RC and RA/LC conditions (see Supplementary Materials Table S1, for individual data).

### 3.1. Words, Pseudowords, and Text Reading

For TEXT reading accuracy, the Wilcoxon signed-rank test showed that stimulation conditions significantly differed in changing reading errors (Z = 2.50, *p* = 0.01, Cohen’s d = 2.58). Following the LA/RC condition, participants displayed fewer errors compared with the RA/LC condition.

No differences between LA/RC and RA/LC conditions emerged for the remaining reading measures: TEXT (Times: Z = 0.56, *p* = 0.58, Cohen’s d = 0.36), HF (Errors: Z = 0.94, *p* = 0.35, Cohen’s d = 0.62; Times: Z = 0.08, *p* = 0.93, Cohen’s d = 0.05), LF (Errors: Z = 1.33, *p* = 0.18, Cohen’s d = 0.93; Times: Z = 0.53, *p* = 0.59, Cohen’s d = 0.34), nor PW (Errors: Z = 0.28, *p* = 0.78, Cohen’s d = 0.18; Times: Z = 0.66, *p* = 0.51, Cohen’s d = 0.43).

### 3.2. Lexical Decision

In the lexical decision task, the Wilcoxon signed-rank test showed stimulation conditions significantly differed in affecting RTs (Z = 2.09, *p* = 0.04, Cohen’s d = 1.76). Following the LA/RC condition, participants showed decreased RTs for word recognition compared with the RA/LC condition.

### 3.3. Phoneme Blending, Working Memory, and Rapid Automatized Naming

Table 2 shows the mean (SD) of the phoneme blending, working memory, and rapid automatized naming tasks for LA/RC and RA/LC conditions (see Supplementary Materials Table S2, for individual data).

No differences were observed between LA/RC and RA/LC conditions for the phoneme blending (Number of Phonemes: Z = 1.30, *p* = 0.19, Cohen’s d = 0.90; Phonemes Times: Z = 0.36, *p* = 0.72, Cohen’s d = 0.23), working memory (Verbal N-back: Z = 1.24, *p* = 0.21, Cohen’s d = 0.85; Visuo-Spatial N-back: Z = 0.77, *p* = 0.44, Cohen’s d = 0.50), and RAN (Letters: Z = 0.46, *p* = 0.65, Cohen’s d = 0.29; Colors: Z = 0.76, *p* = 0.44, Cohen’s d = 0.50).

### 3.4. Coherent Dot Motion

**Figure 3:**
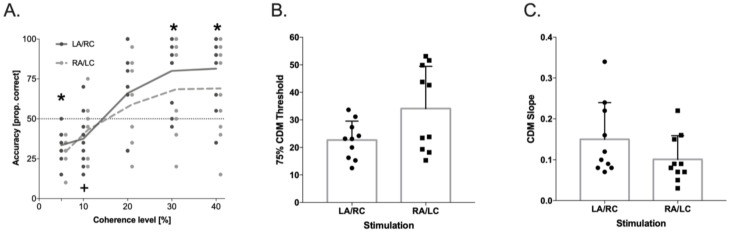
Coherent dot motion (CDM) accuracy (raw data) for the two stimulation conditions; * represents the significant difference from the chance level of 0.25 and from the threshold of 0.5 of accuracy in LA/RC condition. + represents the significant difference from the chance level of 0.25 in RA/LC condition (panel (**A**)), *p* < 0.05 in 75% CDM threshold (panel (**B**)), and *p* < 0.05 in slope values (panel (**C**)), as a function of stimulation condition, obtained from the psychometric curve (logistic) fitted on individual data. Dots represent individual data points.

In the CDM Task, the results showed a significant effect from the Coherence Levels (F(4,36) = 25.88, *p* < 0.001, ηp^2^ = 0.74), and a significant Coherence Levels × Stimulation Condition interaction (F(4,36) = 3.86, *p* < 0.01, ηp^2^ = 0.30; see Figure 3). Post hoc comparisons showed that the accuracy in the LA/RC condition was significantly higher relative to the chance level (i.e., 0.25) starting from the lower coherence level (5%, t(9) = 2.76, *p* < 0.022, Cohens’ d = 1), whereas in the RA/LC condition, the significant difference from the chance level was reached only at the 10% coherence level (t(9) = 3.11, *p* < 0.012, Cohens’ d = 1). In addition, the accuracy in the LA/RC condition was significantly higher than the threshold level (i.e., 0.5), starting at the 30% coherence level (t(9) = 4.62, *p* < 0.001, Cohens’ d = 1.43), while a performance above the threshold was never reached in the RA/LC condition (all *p* > 0.072).

In order to confirm these results by using psychophysics analysis, the individual curves—representing the performance at the different levels of coherence in the CDM task—were fitted by a logistic function [79] for each tDCS stimulation condition. The upper bound was set at 1 and the lower bound at y0 = 0, where y = 0 means that the dots’ motion was never perceived, and y = 1 that it was always perceived. The only free parameters of the function were b (the function slope) and t (the 50% threshold). The resulting logistic function was: y = 1/1 + e − b × (x − t). In this equation, x represents the percentage of motion increment, and y the relative response frequency. The threshold values used for the analysis corresponded to the 75% threshold. Within-subject t-tests were then used to compare the slope and 75% mean thresholds in the CDM task between the two tDCS conditions. The slope for the CDM task was higher (t(9) = 2.983, *p* < 0.02, Cohens’ d = 0.66) in LA/RC (0.15 ± 0.089) than RA/LC (0.10 ± 0.058). The 75% mean threshold was lower (t(9) = −3.49, *p* < 0.01, Cohens’ d = 0.94) in LA/RC (23 ± 7%) than in RA/LC (34 ± 15%). These results demonstrate that motion sensitivity was higher in LA/RC in comparison to RA/LC tDCS stimulation and also that the shape of the respective curves differed significantly, which implies that in LA/RC, the stimuli were processed significantly different than in RA/LC tDCS stimulation.

### 3.5. Attentional Zooming

**Figure 4:**
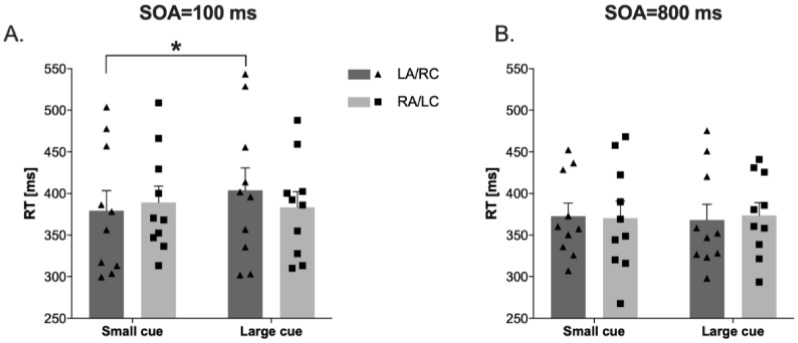
Reaction times (RTs) in attentional zooming task at 100 ms cue-target SOA (panel (**A**)), and at 800 ms cue-target SOA (panel (**B**)) in Left Anodal/Right Cathodal (LA/RC) and Right Anodal/Left Cathodal (RA/LC) conditions. Panel (**A**): RTs in large cue are significantly slower than small cue in LA/RC condition. Dots represent individual data points. * *p* < 0.05

In the attentional zooming task, the results showed a significant effect of Eccentricities (F(2,18) = 6.77, *p* < 0.006, ηp^2^ = 0.43), a Cue × Eccentricities interaction (F(2,18) = 5.58, *p* < 0.013, ηp^2^ = 0.38), and, importantly, a Stimulation condition × Cue × Soa interaction (F(1,9) = 11.29, *p* < 0.008, ηp^2^ = 0.55). Post hoc comparisons showed that at 100 ms SOA in the large cue, the RTs for targets detection in the LA/RC condition are significantly slower than in the small cue (t(9) = −2.50, *p* < 0.034, Cohens’ d = 0.30; see Figure 4).

### 3.6. Correlations between Reading and Neuropsychological Changes

First, changes in TEXT errors, Lexical Decision RTs, Phonemes Times, Letters RAN, Colors RAN, and the CDM task (75% mean thresholds) were calculated by subtracting measures after the LA/RC condition from measures after the RA/LC condition (_ΔRA/LC−LA/RC_). Changes in Number of Phonemes, Verbal N-back, Visuo-Spatial N-back, CDM task (the slope), and Attentional Zooming task (Cue Large minus Cue Small at 100 ms SOA) were calculated by subtracting measures after the RA/LC condition from measures after the LA/RC condition (_ΔLA/RC−RA/LC_).

A significant and positive correlation between TEXT errors _ΔRA/LC-LA/RC_ and the CDM task (75% mean thresholds) _ΔRA/LC-LA/RC_ was found (rho = 0.81, *p* = 0.005), meaning the higher the amount of changes in text errors, the higher the amount of changes in the 75% mean thresholds of the CDM task. Furthermore, a significant and positive correlation between Lexical Decision RTs _ΔRA/LC-LA/RC_ and Visuo-Spatial N-back _ΔLA/RC-RA/LC_ emerged (rho = 0.82, *p* = 0.004), meaning the higher the amount of changes in word recognition speed, the higher the amount of changes in visuo-spatial working memory.

No further significant correlations emerged (*p* > 0.10).

## 4. Discussion

The possibility to modulate reading in individuals with and without dyslexia using transcranial electrical stimulation, such as tDCS, has been documented in the literature [54,55,56,57,58,59,60,61,62]. Nevertheless, the underlying neurocognitive mechanisms mediating reading changes induced by tDCS are still not well understood.

Our results showed significant changes in text reading errors and word recognition speed as a function of specific electrode polarity. Precisely, when tDCS enhanced left neural excitability while decreasing right neural excitability of the temporo-parietal regions (LA/RC condition), text reading errors reduced, and word recognition speed increased as compared to the inverse polarity (RA/LC condition).

These results supported previous evidence that anodal stimulation (increased excitability) in the left temporo-parietal areas combined with concomitant cathodal stimulation (decreased excitability) of contralateral areas can modulate reading, as already showed in typically reading adults and in children and adolescents with dyslexia [62,80].

Intriguingly, we found that by enhancing left neural excitability while decreasing right neural excitability of the temporo-parietal regions, not only was word reading modulated but also MD stream functioning and visuo-spatial attentional zooming changed.

Concerning MD stream functioning, after LA/RC tDCS, participants better discriminated motion direction than after RA/LC tDCS at the lowest motion coherence (5%) and at the highest motion coherences (30 and 40%). These results were confirmed by using psychophysics analyses on the slope, which was higher in the LA/RC than in the RA/LC condition, and on the accuracy threshold, which decreased in the LA/RC compared to the RA/LC condition. Altogether, our results on MD stream functioning indicated better motion sensitivity after reducing the excitability of the right temporo-parietal cortex and enhancing the cortical excitability of the left temporo-parietal cortex.

In addition, our results of the correlations indicated that, after LA/RC tDCS of temporo-parietal regions compared to RA/LC, higher accuracy in the CDM task was related to higher text reading accuracy. In accordance with previous findings supporting the role of MD stream activity in reading skills [45,57,81], word reading change in our participants with dyslexia after anodal stimulation of the left temporo-parietal areas combined with cathodal stimulation of the right temporo-parietal areas was related to better motion perception. The V5/MT+ motion area of the MD stream was found functionally altered in children and adults with dyslexia [82,83], and the contrast responsivity in V5/MT+ was found related with reading measures in a large group of typically reading children [84]. Accordingly, longitudinal and remediation studies [40,41,43,85] showed a causal link between V5/MT+ functioning measured by CDM task and reading acquisition and amelioration.

Concerning visuo-spatial abilities, correlational analyses showed that better visuo-spatial working memory was related to better word recognition after LA/RC tDCS of temporo-parietal regions compared to RA/LC. For visuo-spatial attention, we investigated the time-course of zoom-in and zoom-out functioning [76,78,86] by measuring the detection of visual stimuli that appeared after small and large cues. Following LA/RC stimulation, participants were slower in detecting visual stimuli after the appearance of large cues, as compared to small cues, only at a short cue–target interval. Deficits in zoom-out distributed attention have been shown in children with dyslexia [18,33,82,83,87], and longitudinal and remediation studies [9,40,41,43,85,87] have found a causal link between zoom-out distributed attention and reading acquisition and amelioration. Thus, enhancing the left neural excitability and decreasing the right neural excitability of the temporo-parietal regions apparently affected word reading, motion perception, and attentional zooming. However, neuropsychological, psychophysical, electrophysiological, and functional neuroimaging studies have suggested that global perception and attentional zoom-out, as well as the spatiotemporal integration of moving dots guided by MD stream functioning mainly recruit the right temporo-parietal junction, while the homologous area in the left hemisphere specifically processes local details and attentional zoom-in [88,89,90]. Moreover, greater right prefrontal activation during reading and white-matter organization of the right superior longitudinal fasciculus (including arcuate fasciculus) predicted reading acquisition in dyslexia [91]. The role of the right temporo-parietal areas in dyslexia was confirmed by functional neuroimaging studies in children with dyslexia, demonstrating reduced brain activity in these areas [92]. Strictly, functional and structural brain abnormalities in pre-reading children at risk for dyslexia were found in the right parietal lobe [93]. Furthermore, behavioral and psychophysics evidence supported the contribution of the right temporo-parietal attentional deficits in dyslexia [32,77,94]. Considering these previously described findings on right temporo-parietal deficits in dyslexia, a possible alternative interpretation of positive effects on motion perception and attentional zoom-out after LA/RC tDCS could be the excitability reduction induced by the cathodal stimulation of the right temporo-parietal attentional network in conjunction with the increased excitability induced by the anodal stimulation of the left temporo-parietal reading network. This suggests that cathodal stimulation over the right regions could reduce “neural-noise”. In accordance with Hancock et al. [95], a causal link between increased neural noise and reading disorders has been proposed. It has been hypothesized that in dyslexia, an excessive excitability of “distal” spontaneous neural firing—combined with an anomalous exogenous and endogenous oscillatory synchronization—may disrupt temporal sensory processing and perceptual noise-exclusion mechanisms that are crucial for efficient audiovisual processing and cross-modal binding.

In particular, hyperexcitability in local networks—induced by an enhanced glutaminergic activation [96]—can hamper excitation/inhibition balance and the precise timing of neural activity, affecting performance in a performance/arousal inverted U-shape relationship [95]. Although the neural noise hypothesis of dyslexia [95] was mainly targeted on the left phonological and orthographic brain networks, it could not be excluded that the beneficial effect we found on reading, visuo-spatial attention, and MD stream functioning was more the result of an inhibitory effect over the right temporo-parietal cortex than of the excitatory effect over the left temporo-parietal cortex. Taken together, our results may be interpreted in light of a whole-brain excitation/inhibition unbalance in which not only the left phonological network, but also the right attentional network is affected [32,77,93,94]. By decreasing the cortical excitability in the right temporo-parietal areas and increasing the cortical excitability in the contralateral areas, we changed the excitation–inhibition neural balance activity within the temporo-parietal regions. Since unilateral right posterior temporal gyrus activation has been described during sentence and text processing in typical readers [97], our participants with dyslexia could have altered excitability of the right hemisphere and the LA/RC montage could have rebalanced this alteration by reducing the number of errors in text reading compared to the RA/LC tDCS condition. Similarly, the significant effect found in motion perception (i.e., CDM accuracy, slope, and threshold change) after the LA/RC tDCS condition could be explained by a reduction in the cortical excitability of the right temporo-parietal areas and by an increase in the cortical excitability of the left temporo-parietal areas. Indeed, these regions of the right hemisphere seem to be highly involved in zoom-out distributed attention, in the global perception of visual scene [88], and, specifically for our task, in the spatiotemporal integration required to perceive coherently moving dots. Moreover, the observed slowdown of visual detection after a large cue and speeding up the visual detection after a small cue in the attentional zooming task could be related to difficulties in zooming-in following the enhanced excitability of the left temporo-parietal regions and to the facilitation of zooming-out following the decreased excitability of the contralateral areas. While the right temporo-parietal areas of the human brain are more involved in the attention zoom-out [88], the left areas seemed to be particularly involved the zoom-in of the attentional focus [98].

Although, to the best of our knowledge, this study is the first attempt to clarify the relation between brain modulation and changes in reading, phonological processes, visual attention, and MD stream functioning in dyslexia, our results are derived from a small group of children and adolescents with dyslexia and should be taken with caution until more studies replicate them on larger groups.

Further studies are needed also to clarify the neurophysiological effects of tDCS on the dyslexic brain, by means of in vitro studies or by the combination of electrophysiological techniques. As documented in other developmental disorders [99], the effect of brain stimulation may differ from what is expected in a typically developing brain. Studies aimed at clarifying the effect of non-invasive brain stimulation on the cortical excitability/inhibitory unbalance may increase our understanding of the modulatory effect of tDCS in dyslexia.

## 5. Conclusions

In conclusion, by increasing the neural excitability of the left temporo-parietal areas while reducing the neural excitability of the right temporo-parietal areas in children and adolescents with dyslexia, we found that: (i) word text accuracy and word recognition improved, (ii) visuo-spatial attentional focusing reduced, and (iii) motion perception enhanced.

Our findings demonstrated that reading and domain-general neurocognitive functions such as visuo-spatial attention and MD stream functioning are affected by tDCS and that those changes are polarity-dependent.

## Figures and Tables

**Table 1:** Mean (SD) of each reading measure and lexical decision task for LA/RC and RA/LC conditions.

		LA/RC	RA/LC
Reading Measures		M (SD)	M (SD)
TEXT	% Errors ^1^	4.78 (4.16)	6.89 (5.47) **
	Times ^2^	41.90 (23.27)	40.72 (24.66)
High Frequency words	% Errors ^1^	6.75 (8.74)	9.00 (15.64)
	Times ^3^	18.40 (10.99)	20.00 (16.11)
Low Frequency words	% Errors ^1^	12.25 (12.10)	15.75 (18.52)
	Times ^3^	28.40 (16.04)	32.80 (26.86)
Pseudo- words	% Errors ^1^	20.75 (26.90)	20.00 (21.21)
	Times ^3^	36.30 (15.95)	35.66 (16.91)
Lexical Decision	Reaction Times ^3^	1.34 (0.40)	1.50 (0.42) *

Notes: ^1^ percentage (%) of errors, calculated as total number of errors/total number of words × 100; ^2^ s/syllables × 100; ^3^ in s. LA/RC = Left Anodal/Right Cathodal; RA/LC = Right Anodal/Left Cathodal. Significant difference between conditions: * *p* < 0.05; ** *p* < 0.01.

**Table 2:** Mean (SD) of the phoneme blending, working memory, and rapid automatized naming tasks for LA/RC and RA/LC conditions.

		LA/RC	RA/LC
Neuropsychological Tasks		M (SD)	M (SD)
**Phoneme Blending**	Accuracy ^1^	62.80 (16.46)	65.00 (15.58)
	Times ^2^	22.33 (8.55)	23.91 (10.46)
**N-back**	Verbal ^3^	2.56 (0.60)	2.73 (0.45)
	Visuo-Spatial ^3^	2.78 (0.73)	2.93 (0.70)
**RAN**	Letters ^2^	3.75 (1.14)	5.03 (1.34)
	Colors ^2^	4.82 (1.11)	3.84 (0.76)

Notes: ^1^ Number of phonemes; ^2^ in s; ^3^ index of efficiency. RAN = Rapid Automatized Naming; LA/RC = Left Anodal/Right Cathodal; RA/LC = Right Anodal/Left Cathodal.

## Supplementary Materials

The following are available online at https://www.mdpi.com/2076-3425/11/2/263/s1, Table S1: Individual data of the reading measures and lexical decision task in LA/RC and RA/LC conditions, Table S2: Individual data of the phoneme blending, working memory and rapid automatized naming tasks in LA/RC and RA/LC conditions.
