# Supplementary material for: Beyond Reading Modulation: Temporo-Parietal tDCS Alters Visuo-Spatial Attention and Motion Perception in Dyslexia
Source: Brain Sci. 2021 Feb 19;11(2):263. doi: 10.3390/brainsci11020263 (PMC7922381; doi:10.3390/brainsci11020263)
Supplement: Supplementary file 1 [file brainsci-11-00263-s001.pdf]

Table S1. Individual data of the reading measures and lexical decision task in LA/RC and RA/LC conditions.

ID	TEXT				High Frequency Words				Low Frequency Words				Pseudowords				Lexical Decision	
	% Errors[1]		Times[2]		% Errors[1]		Times[3]		% Errors[1]		Times[3]		% Errors[1]		Times[3]		Times[3]	
	LA/RC	RA/LC	LA/RC	RA/LC	LA/RC	RA/LC	LA/RC	RA/LC	LA/RC	RA/LC	LA/RC	RA/LC	LA/RC	RA/LC	LA/RC	RA/LC	LA/RC	RA/LC
**1**	1.90	3.00	29.00	32.00	0.00	0.00	10	10	7.50	5.00	18	21	15.00	10.00	35	30	1.33	1.28
**2**	6.50	5.60	38.20	38.60	5.00	5.00	13	16	17.5	20.00	32	29	12.50	10.00	30	27	1.40	1.71
**3**	0.00	2.70	27.20	29.70	0.00	0.00	10	11	0.00	5.00	14	14	0.00	5.00	20	18.6	1.54	1.53
**4**	0.00	1.30	19.00	19.50	0.00	5.00	9	11	0.00	0.00	9	13	0.00	0.00	14	16	0.72	0.78
**5**	1.60	2.60	21.00	25.20	0.00	0.00	12	12	15.00	0.00	16	13	5.00	0.00	26	28	1.10	1.30
**6**	10.70	14.20	54.00	31.46	25.00	50.00	41	62	40.00	60.00	55	100	90.00	70.00	53	72	1.04	1.37
**7**	7.80	10.90	72.40	58.00	17.50	0.00	25	20	15.00	22.50	42	38	17.50	30.00	50	41	1.35	1.88
**8**	2.00	1.90	21.50	19.80	2.50	0.00	14	10	0.00	0.00	15	14	5.00	20.00	27	33	1.20	1.19
**9**	7.00	11.40	86.00	101.00	5.00	12.50	33	31	10.00	22.50	46	50	27.50	20.00	64	55	2.25	2.27
**10**	10.40	15.30	50.70	51.90	12.50	17.50	17	17	17.50	22.50	37	36	35.00	35.00	44	36	1.47	1.72

Notes: [1] percentage (%) of errors, calculated as total number of errors/total number of words x 100; [2] seconds/syllables x 100; [3] seconds. LA/RC = Left Anodal/Right Cathodal; RA/LC = Right Anodal/Left Cathodal.

Table S2. Individual data of the phoneme blending, working memory and rapid automatized naming tasks in LA/RC and RA/LC conditions.

ID	**Phoneme Blending**				**N-back**				**RAN**			
	Accuracy[1]		Times[2]		Verbal[3]		Visuo-Spatial[3]		Letters[2]		Colours[2]	
	LA/RC	RA/LC	LA/RC	RA/LC	LA/RC	RA/LC	LA/RC	RA/LC	LA/RC	RA/LC	LA/RC	RA/LC
**1**	71	75	21.00	11.60	2.38	2.72	2.00	2.54	4.65	3.60	5.30	6.25
**2**	57	58	18.91	20.50	3.36	3.35	3.68	2.75	4.05	3.90	3.85	5.60
**3**	64	73	28.90	19.50	2.61	3.64	3.40	3.76	2.30	2.80	2.90	3.45
**4**	76	68	13.20	19.00	3.50	2.72	3.63	3.70	3.10	4.10	4.75	4.45
**5**	71	73	21.81	30.45	2.33	2.40	2.33	2.33	3.51	5.00	4.61	4.95
**6**	63	69	26.70	38.33	2.60	2.33	2.71	2.66	5.15	4.35	5.65	4.32
**7**	19	23	39.26	44.75	2.33	2.71	1.75	1.55	3.10	3.40	6.80	7.75
**8**	74	74	12.70	18.10	2.64	2.64	2.42	3.26	2.40	2.70	4.05	4.40
**9**	63	71	27.90	18.20	1.30	2.13	2.27	3.50	3.45	4.75	5.80	5.70
**10**	70	66	12.91	18.72	2.58	2.66	3.57	3.28	5.75	3.80	4.50	3.40

Notes: [1] Number of Phonemes; [2] in seconds; [3] index of efficiency. RAN = Rapid Automatized Naming; LA/RC = Left Anodal/Right Cathodal; RA/LC = Right Anodal/Left Cathodal.
